# SHBG as a Marker of NAFLD and Metabolic Impairments in Women Referred for Oligomenorrhea and/or Hirsutism and in Women With Sexual Dysfunction

**DOI:** 10.3389/fendo.2021.641446

**Published:** 2021-03-29

**Authors:** Vincenza Di Stasi, Elisa Maseroli, Giulia Rastrelli, Irene Scavello, Sarah Cipriani, Tommaso Todisco, Sara Marchiani, Flavia Sorbi, Massimiliano Fambrini, Felice Petraglia, Mario Maggi, Linda Vignozzi

**Affiliations:** ^1^ Andrology, Women’s Endocrinology and Gender Incongruence Unit, Department of Experimental Clinical and Biomedical Sciences “Mario Serio,” University of Florence, Florence, Italy; ^2^ Gynecology Unit, Department of Biomedical, Experimental and Clinical Sciences “Mario Serio,” University of Florence, Florence, Italy; ^3^ Endocrinology Unit, Department of Experimental Clinical and Biomedical Sciences “Mario Serio,” University of Florence, Florence, Italy; ^4^ I.N.B.B. (Istituto Nazionale Biostrutture e Biosistemi), Rome, Italy

**Keywords:** sex hormone binding globulin (SHBG), non-alcoholic fatty liver disease (NAFLD), polycystic ovary syndrome (PCOS), metabolic syndrome, female sexual dysfunction

## Abstract

PCOS is one of the most common endocrine disorders and NAFLD is one of its most dangerous metabolic consequences. The diagnosis of NAFLD is not a practical task and the condition is at risk of being overlooked. The use of simpler but still reliable surrogate markers is necessary to identify women with a high likelihood of NAFLD. The aim of this study was to evaluate the clinical correlates of NAFLD Liver Fat Score (NAFLD-LFS) in women with oligomenorrhea and/or hirsutism. Furthermore, the study aimed to evaluate whether, among the hormonal parameters evaluated in such women, possible hallmarks of NAFLD may be identified. To this purpose, 66 women who attended our Outpatient Clinic for oligomenorrhea and/or hyperandrogenism were included in the study. In order to validate the results obtained in the first cohort, a second independent sample of 233 women evaluated for female sexual dysfunction (FSD) was analyzed. In cohort 1, NAFLD-LFS positively correlated with metabolic and inflammatory parameters. Among the hormone parameters, NAFLD-LFS showed no significant relationships with androgens but a significant negative correlation with SHBG (p<0.0001) that therefore appeared as a candidate hallmark for pathologic NAFLD-LFS. The ROC analysis showed a significant accuracy (81.1%, C.I.69.1-93.0, p <0.0001) for SHBG in identifying women with a pathological NAFLD-LFS. In particular, a SHBG 33.4 nmol/l was recognized as the best threshold, with a sensitivity of 73.3% and a specificity of 70.7%. In order to validate this SHBG as a marker of metabolic impairment possible related with the presence of NAFLD, we tested this threshold in cohort 2. FSD women with SHBG <33.4 nmol/l had worse metabolic parameters than women with SHBG ≥33.4 nmol/l and a significantly higher NAFLD-LFS even after adjusting for confounders (B=4.18 [2.05; 6.31], p=0.001). In conclusion, this study provides a new evidence in the diagnostic process of NAFLD, showing that the measurement of SHBG, which is routinely assessed in the workup of women referred for possible PCOS, could identify women at higher metabolic risk, thus detecting those who may deserve further targeted diagnostic assessment.

## Introduction

Non-alcoholic fatty liver disease (NAFLD) is characterized by a spectrum of disorders ranging from the simple fatty liver to non-alcoholic steatohepatitis (NASH), with increasing fibrosis leading to cirrhosis (1–4). Nowadays, NAFLD is considered the hepatic hallmark of insulin resistance in several metabolic disorders. Hence, recognizing NAFLD may be of pivotal importance because it increased the risk of developing not only hepatic but also extrahepatic diseases, such as cardiovascular disease (CVD), chronic kidney disease (CKD) and type 2 diabetes mellitus (T2DM) (5–7). Growing evidence indicates a high prevalence of NAFLD in women with polycystic ovary syndrome (PCOS). In fact, in PCOS, NAFLD ranges from 34% to 70% as compared with a 14–34% in the female general population (8–15). This emerging association between NAFLD and PCOS was substantiated by evidence of common pathogenic mechanisms between the two conditions (16, 17). Recent studies confirmed that both insulin resistance and hyperandrogenism are key players of liver damage in women with PCOS, with a major role of the second one in non-obese patients (18, 19). PCOS, one of the most common endocrine disorders, affects 5-10% of reproductive-aged women (20), presenting not only a reproductive, but also an oncological and a cardio-metabolic burden. Among these, NAFLD is often overlooked. Therefore, due to this high frequency and its key consequences, the evaluation of hepatic health should be mandatory in patients with PCOS. However, the diagnosis of NAFLD is not an easy task. Liver biopsy remains the gold standard; however, it may not be considered the first line procedure in a prevalent condition such as PCOS. Therefore, the use of simpler, but still reliable, surrogate markers is necessary to identify women with a high likelihood of NAFLD. To this purpose, several algorithms, based on clinical and biochemical easily available information, have been introduced (21, 22). The best-validated steatosis algorithms are the SteatoTest^®^, the Fatty Liver Index (FLI) and the NAFLD Liver Fat Score. All these algorithms have been validated in the general population or in severe obese populations and variably predict hepatic and cardio-metabolic outcomes/mortality (21, 23).

In the last years, the FLI and the NAFLD –LFS algorithms were applied in large cohorts of patients showing great accuracy as markers of liver damage, in particular in metabolically susceptible populations (13, 24–26). In addition, a very recent study, comparing four non-invasive NAFLD score, i.e. FLI, NAFLD-LFS, Hepatic Steatosis Index (HIS) and Lipid Accumulation Product (LAP), concluded that, whereas FLI is the most accurate in a population-based setting, NAFLD- LFS performs better in the high-risk subjects (27).

The aim of the present study was to evaluate the clinical and biochemical correlates of NAFLD-LFS in women who refer to an outpatient endocrinology clinic for oligomenorrhea and/or hirsutism and thus undergoing further investigations for PCOS diagnosis. Furthermore, since NAFLD-LFS is more convenient than liver biopsy but still impractical for routine clinical practice, the study aimed to evaluate whether, among the hormonal parameters, routinely used for the diagnostic workup of PCOS, there are any that better predicts a pathological NAFLD-LFS. Essentially, we found that sex hormone binding globulin (SHBG) below 33.4 nmol/l was able to predict a high risk for NAFLD in women consulting for PCOS, thus suggesting it as a possible biochemical hallmark of this hepatic disease. Therefore, in the second part of the study we tested this cut-off in a larger cohort of women referring to our Unit for PCOS-unrelated reasons, such as sexual difficulties.

## Materials and Methods

### Study Design

This study was designed as a cross-sectional prospective study including two cohorts of women attending our outpatient clinics. Data were retrospectively collected by revising the medical records.

### Patients’ Recruitment (Cohort 1 and Cohort 2)

The first cohort of this study included a consecutive series of 66 women who attended the Andrology, Women’s Endocrinology and Gender Incongruence Outpatient Clinic at the University of Florence (Florence, Italy) seeking medical care for oligomenorrhea and/or clinical hyperandrogenism and, therefore, evaluated for possible PCOS. The study protocol was in accordance with the Declaration of Helsinki and was approved by the local ethics committee (protocol PCOSFLOWMETS-12/811 OSS, Careggi Hospital, Florence, Italy). Informed consent was obtained before the initiation of any clinical procedures (a parent’s consent was obtained for underage patients). PCOS was diagnosed according to the Rotterdam Criteria (28, 29). Exclusion criteria were the differential diagnosis of PCOS (thyroid diseases, hyperprolactinemia, non-classical congenital adrenal hyperplasia, acromegaly and Cushing disease), uncontrolled psychiatric disorders and inability to provide study consent. At the first visit, demographic and clinical data were collected as part of routine practice, including information on menstrual cycle, sexual life, habit to perform physical activity, medications used and associated medical conditions. “Physical activity” is a dichotomous variable (yes/no) in which “yes” indicates that the patients performed physical activity (of any kind) and “no” indicates that the patients did not perform any type of physical activity. “Hours of physical activity/week” is a categorical variable including 4 levels: No physical activity - 1-3 hours/week - 4-6 hours/week - more than 7 hours for week. Patients also underwent a physical examination with measurement of body weight, height, body mass index (BMI), waist circumference, bioimpedance analysis, systolic and diastolic blood pressure. Hirsutism was evaluated using the modified Ferriman Gallwey (mFG) Score (30). Hirsutism was defined by a mFG score ≥ 8 for caucasian women (100% of the analytical sample). Polycystic Ovarian Morphology (PCOM) was defined according to the criteria of *Androgen Excess and Polycystic Ovary Syndrome Society* (31). Specifically, new ultrasound machines (transducers with frequencies ≥8 mHz) allow diagnosis of PCOM in patients having at least 20 small follicles (2 to 9 mm) in the whole ovary while ovarian size at 10 mL remains the threshold for the definition of increased ovary size (preferred criterion when using transducer frequencies <8 mHz) (31, 32).

In order to validate the results obtained in the first cohort, a second independent sample was analyzed (Cohort 2). Cohort 2 included 233 consecutive patients who attended the Andrology, Women’s Endocrinology and Gender Incongruence Outpatient Clinic seeking medical care for female sexual dysfunction (FSD). These women did not take drugs that could alter SHBG levels, such as antiepileptics and estroprogestins. Informed consent was obtained before the initiation of any clinical procedures. Exclusion criteria were history of drug or alcohol abuse and a diagnosis of uncontrolled or unstable disease. At the first visit, demographic and clinical data were collected as part of routine practice, including information on menopause, medications used, and associated medical conditions. Previous diagnoses of mental disorders were assessed using the *Diagnostic and Statistical Manual of Mental Disorders, Fifth Edition* criteria (33). Patients also underwent a physical examination with measurement of body weight, height, BMI, waist circumference, bioimpedance analysis, systolic and diastolic blood pressure. The assessment of NAFLD-LFS was possible in 26 patients (11.2% of total cohort). All patients underwent metabolic assessment and a Color Doppler Ultrasound (CDU) examination of clitoral vascularization.

### Biochemical Parameters

In patients from both the cohorts, blood samples were drawn in the morning after an overnight fast in early follicular phase (in pre-menopausal women) for the measurement of metabolic and hormonal parameters. Among the metabolic parameters we measured: blood glucose (using the glucose hexokinase method; Dimension Vista 1500, Siemens Medical Solutions USA, Malvern, PA, USA), total cholesterol, high-density lipoprotein cholesterol, and triglycerides (using the automated enzymatic colorimetric method; Dimension Vista 1500), insulin levels (using an electro-chemiluminescence immunoassay; Roche Diagnostics, Mannheim, Germany), glycated hemoglobin levels (using high-performance liquid chromatography; Variant II, Biorad Laboratories, Hercules, CA, USA), AST and ALT (COBAS 8000, Roche). Low-density lipoprotein (LDL) cholesterol was estimated indirectly by the Friedewald formula, unless triglycerides were >400 mg/dl (34).

In addition, the following hormones were measured: LH, Follicle- Stimulating Hormone (FSH), Estradiol (E), prolactin, thyroid stimulating hormone (TSH), FT3, FT4 (using the chemiluminescence method; DIMENSION VISTA ^®^ System, Siemens), testosterone (using the chemiluminescence method; CENTAUR, Siemens), 17 alpha-OH-progesterone (using the RadioImmunoAssay method; DIASOURCE, Belgium), delta 4-androstenedione (using RadioImmunoAssay method; BECKMAN COULTER), dehydroepiandrosterone sulfate, DHEAS (using the electro-chemiluminescence immunoassay; COBAS, ROCHE, Germany), SHBG (using the electro-chemiluminescence immunoassay; COBAS, ROCHE, Germany) and anti-Mullerian hormone, AMH (using the chemiluminescence method; BECKMAN COULTER). Free androgen index (FAI) was calculated as the total testosterone to SHBG ratio and then multiplied by 100. HOMA IR was calculated as (fasting plasma glucose*insulin/405) where glucose was expressed in mg/dl and insulin in mU/L (35).

### Non-Alcoholic Fatty Liver Disease Assessment

The risk of being affected by NAFLD was estimated by the NAFLD Liver Fat Score (NAFLD-LFS) (36), according to the following formula:

NAFLD-LFS: -2.89 + 1.18 * metabolic syndrome (yes = 1/no = 0) + 0.45* type 2 diabetes (yes =2/no=0) + 0.15 * insulin (mU/L) + 0.04 *AST (U/L) - 0.94 * AST/ALT

Metabolic syndrome (MetS) was defined according to criteria of the International Diabetes Federation (IDF) (37). In addition, the IDF adult criteria can be used for adolescents aged ≥ 16 years, while a modified version of these criteria can be applied to those aged 10 to < 16 years (use 90th percentile cutoff point for waist and < 40 mg/dl of HDL) (38).

NAFLD-LFS above -0.640 predicted NAFLD with sensitivity of 86% and specificity of 71% (36).

Other possible causes of liver steatosis were ruled out by history taking.

### Color Doppler Ultrasound Assessment

As for clinical practice, CDU was performed only in cohort 2 by an experienced operator blinded to the clinical data using the MyLabClass-C sonography system (Esaote SpA, Genova, Italy); a linear transducer (LA523, 6e13 MHz) was used. All women were scanned in a quiet room with consistent conditions of heating and lighting to decrease the impact of external factors on blood flow. For premenopausal women, CDU was carried out during the early follicular phase of the menstrual cycle (days 3-5). CDU was performed according to a previously published operating procedure (39–50). Briefly, the exam was performed after 12 hours of sexual abstention (sexual intercourse or masturbation), and immediately after bladder voiding. Patients were scanned in the lithotomy position, with a good quantity of sonographic jelly to avoid interference from air and without applying any significant pressure on the genital tissues, to minimize possible artifacts (51, 52). A cross-section of the clitoris was obtained by placing the probe transversally at the top of the vulva; this plane allows for easy localization of the cavernous arteries, which appear well defined at the center of each clitoral body (51, 52). When adequate Doppler signals were detected, pulse-wave Doppler mode was activated and blood flow velocity waveforms were recorded, with automatic computation of the PI. The PI represents the difference between the peak systolic and the end-diastolic flows divided by the mean maximum flow velocity (53). Because it characterizes the shape of the spectral waveform, it is independent of the probe angle to the vessel (54). At least three similar sequential waveforms were sampled for each cavernous artery to define a mean PI value.

### Statistical Analysis

Data were expressed as mean ± SD when normally distributed and as median (quartile) for parameters with non-normal distribution, unless otherwise specified. Furthermore, categorical variables were reported as number and percentage. Correlations were assessed using the Spearman method. Significant correlations at univariate analysis were tested at multivariate analysis after adjusting for confounding factors. Linear regression was applied for multivariate analysis. ROC curve analysis was used for the evaluation of the accuracy of SHBG in detecting the risk to have NAFLD, and the coordinates of the ROC curve have been evaluated for the identification of a possible threshold value. All statistical analyses were performed using SPSS 26.0 for Windows (SPSS Inc, Chicago, IL, USA).

## Results

**Table 1** lists the main characteristics of the two samples and the comparisons for the available variables. In the first cohort, 55 out of 66 patients were diagnosed with PCOS (according Rotterdam criteria). Nine patients were diagnosed such as “not PCOS” (i.e. only oligomenorrhea or hyperandrogenism) and two patients had not a definitive diagnosis, at the time of the study analysis. At univariate analysis, NAFLD-LFS was positively correlated with BMI, waist circumference, fasting plasma glucose (FPG), insulin, glycated hemoglobin, HOMA index, HDL cholesterol, triglycerides, blood pressure (SBP and DBP), fat mass (FM), erythrocyte sedimentation rate (ESR), C-reactive protein (CRP), white blood cells (WBC) and FAI. Conversely, NAFLD-LFS negatively correlated with the time spent weekly for physical activity. Accordingly, women reporting regular physical activity had significantly lower NAFLD-LFS than physically inactive women did (-0.74 vs.-1.80 p=0.015). Among the hormone parameters, NAFLD-LFS showed a significant negative correlation only with SHBG, while there were no correlations between NAFLD-LFS with testosterone and other androgens (**Table 2**). Considering that the NAFLD-LFS algorithm includes, among its factors, waist circumference, SBP and DBP and that BMI largely overlaps with waist circumference measurement, SHBG was then identified as the candidate hallmark for pathologic NAFLD-LFS.

**Table 1:** Characteristics of the two samples.

	Cohort 1 (66 women with oligomenorrhea and/or hyperandrogenism)	Cohort 2 (233 women with FSD)	p-Value
**Age (years)**	20.0 [17.8–25.0]	48.0 [36.0–56.0]	**<0.0001**
**BMI (kg/m^2^)**	23.7 [21.0–29.0]	24.4 [21.4–29.0]	0.462
**Waist circumference (cm)**	85.0 [74.0–99.3]	92.0 [84.0–103.2]	**0.001**
**FPG (mg/dl)**	85.5 [81.0–91.3]	90.0 [83.8–98.0]	**0.001**
**Insulin (mU/L)**	10.0 [6.2-15.5]	7.5 [5.2–14.2]	0.121
**Glycated Hemoglobin (mmol/mol)**	33.0 [31.0–35.0]	37.0 [34.0–41.0]	**<0.001**
**HOMA IR**	2.2 [1.3–3.3]	1.6 [1.1–3.3]	0.149
**Total cholesterol (mg/dl)**	166.9 ± 30.1	203.2 ± 41.1	**<0.0001**
**HDL cholesterol (mg/dl)**	56.8 ± 13.2	61.6 ± 16.0	**0.036**
**Triglycerides (mg/dl)**	72.0 [52.8–93.0]	79.0 [56–119.5]	0.088
**LDL cholesterol (mg/dl)**	89.5 [69.0–116.3]	119.0 [99.0–144]	**<0.001**
**SBP (mm Hg)**	113.5 [100.0–120.0]	120.0 [110.0–130.0]	**<0.0001**
**DBP (mm Hg)**	70.0 [63.8–80.0]	75.0 [70.0–80.0]	**0.041**
**FM (kg)**	15.7 [10.6–24.2]	18.9 [14.9–27.5]	0.125
**FFM (kg)**	47.3 [45.2–51.1]	43.1 [41.7–45.8]	**<0.0001**
**PCOS (%)**	83.3 [n = 55]	—	—
**Not PCOS (%)**	13.6 [n = 9]	—	—
**Patients without definitive diagnosis of PCOS (%)**	3.0 [n = 2]	—	—
**Physical activity (%)**	48.5 [n = 32]	30.5 [n = 71]	0.060
**Menopause (%)**	—	46.8 [109]	
**Sexually active (%)**	54.5 [n = 36]	—	—
**Smoking habit (%)**	6.1 [n = 4]	21.0 [n = 49]	0.467
**ESR (mm/h)**	16.0 [6.0–25.5]	—	—
**Uric acid (mg/dl)**	4.5 ± 1.0	—	—
**CRP (mg/dl)**	0.4 [0.1–0.9]	—	—
**Ferritin (ng/ml)**	30.0 [16.5–47.5]	—	—
**WBC (mm3)**	6.8 ± 2.1	—	—
**LH (U/L)**	7.3 [4.0–12.1]	11.5 [5.3–30.4]	**<0.0001**
**FSH (U/L)**	5.4 [4.3–6.4]	18.4 [7.0–70.2]	**<0.0001**
**Estradiol (pg/ml)**	38.5 [26.8–59.3]	28.0 [13.6–52.0]	**0.035**
**Prolactin (ng/ml)**	14.6 [8.8–17.8]	9.2 [7.2–15.3]	**0.001**
**Testosterone (nmol/L)**	1.5 [1.0–2.2]	0.8 [0.5–1.4]	**<0.0001**
**SHBG (nmol/L)**	36.1 [27.4–62.7]	58.7 [41.2–82.8]	**<0.0001**
**FAI**	3.6 [1.9–7.0]	1.5 [0.8–2.5]	**<0.0001**
**Androstenedione nmol/L)**	7.6 [5.4–11.8]	4.4 [2.5–6.5]	**<0.0001**
**DHEAS (µmol/L)**	6.4 [4.9–8.2]	2.8 [1.6–4.4]	**<0.0001**
**AMH (ng/ml)**	7.0 [4.4–10.2]	0.2 [0.1–2.4]	**<0.0001**

Data are expressed as mean ± SD when normally distributed, median (quartile) when not normally distributed, and percentage when categorical.

Differences in not normally distributed continuous variables were assessed by Mann–Whitney U test for comparison between the two groups. Differences in normally distributed continuous variables were assessed by unpaired T test. Differences in categorical variables were assessed by Chi squared test.

BMI, Body Mass Index; FPG, Fasting Plasma Glucose; SBP, Systolic Blood Pressure; DBP, Diastolic Blood Pressure; PCOS, Polycystic Ovary Syndrome; ESR, Erythrocyte Sedimentation Rate; CRP, C-Reactive Protein; WBC, White Blood Cells; LH, Luteinizing Hormone; FSH, Follicle Stimulating Hormone; SHBG, Sex Hormone Binding Globulin; FAI, Free Androgen Index; DHEAS, Dehydro-epiandrosterone Sulfate; AMH, Anti Mullerian Hormone.

The bold values are statistically significant values.

**Table 2:** Associations between NAFLD-LFS and clinical and laboratory parameters.

NAFLD-LFS			
	n	Univariate analysis*	
		r	P value
**Age (years)**	66	0.133	0.288
**BMI (kg/m^2^)**	64	0.657	**<0.0001**
**Waist circumference (cm)**	55	0.598	**<0.0001**
**FPG (mg/dl)**	66	0.427	**<0.0001**
**Insulin (mu/L)**	66	0.850	**<0.0001**
**Glycated hemoglobin (mmol/mol)**	57	0.275	**0.035**
**HOMA IR**	59	0.867	**<0.0001**
**Total cholesterol (mg/dl)**	66	0.037	0.768
**HDL cholesterol (mg/dl)**	66	−0.286	**0.022**
**Triglycerides (mg/dl)**	66	0.333	**0.006**
**LDL cholesterol (mg/dl)**	61	−0.029	0.822
**SBP (mm Hg)**	66	0.404	**0.001**
**DBP (mm Hg)**	66	0.473	**<0.0001**
**FM (kg)**	42	0.579	**<0.0001**
**FFM (kg)**	42	0.260	0.097
**Physical activity**	65	−0.271	**0.029**
**Hours of physical activity/week**	65	−0.287	**0.020**
**ESR (mm/h)**	49	0.527	**<0.0001**
**Uric acid (mg/dl)**	45	0.180	0.237
**CRP (mg/dl)**	47	0.375	**0.009**
**Ferritin (ng/ml)**	53	0.236	0.088
**WBC (mm3)**	59	0.317	**0.014**
**LH (U/L)**	65	0.195	0.120
**FSH (U/L)**	65	0.066	0.601
**Estradiol (pg/ml)**	54	0.033	0.815
**Prolactin (ng/ml)**	62	0.168	0.192
**Testosterone (nmol/L)**	63	0.127	0.320
**SHBG (nmol/L)**	56	−0.557	**<0.0001**
**FAI**	55	0.487	**<0.0001**
**Androstenedione (nmol/L)**	60	0.224	0.085
**DHEAS (µmol/L)**	62	−0.188	0.142
**AMH (ng/ml)**	59	−0.162	0.220

*Unadjusted correlation coefficients (r) and levels of significance (P) were derived from Spearman analysis.

BMI, Body Mass Index; FPG, Fasting Plasma Glucose; SBP, Systolic Blood Pressure; DBP, Diastolic Blood Pressure; FM, Fat Mass; FFM, Free Fat Mass; ESR, Erythrocyte Sedimentation Rate; CRP, C-Reactive Protein; WBC, White Blood Cells; LH, Luteinizing Hormone; FSH, Follicle Stimulating Hormone; SHBG, Sex Hormone Binding Globulin; FAI, Free Androgen Index; DHEAS, Dehydro-epiandrosterone Sulfate; AMH, Anti Mullerian Hormone.

The bold values are statistically significant values.

**Figure 1:**
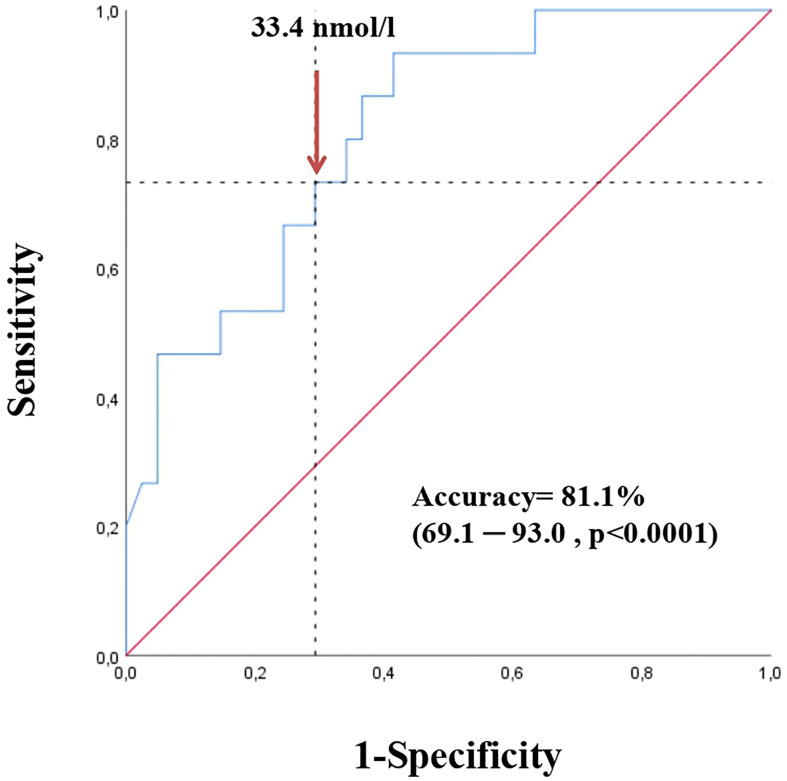
Receiver operating characteristic (ROC) curve for SHBG in detecting NAFLD risk according to NAFLD-LFS (values > -0.640 predict NAFLD with sensitivity of 86% and specificity of 71 %) in the first cohort.

**Figure 2:**
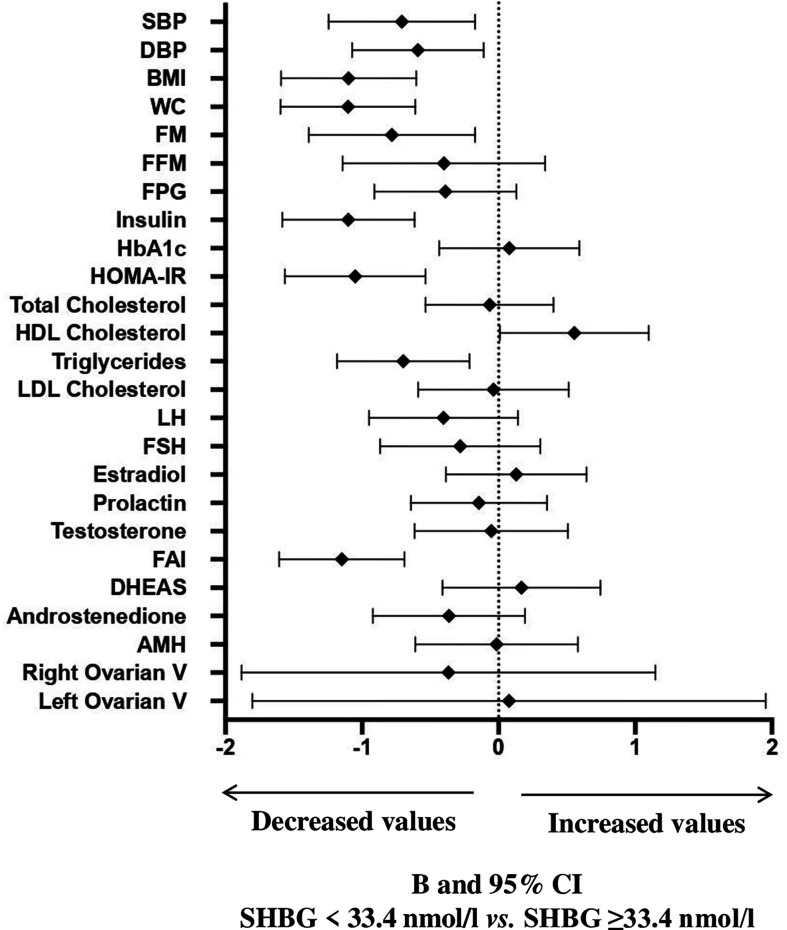
B and 95% confidence interval (CI) for several metabolic and hormonal parameters as a function of SHBG <33.4 nmol/L as compared with SHBG ≥33.4 nmol/L in Cohort 1. Data are expressed as number of standard deviations from the mean value. The standardized values are based on log-transformed parameters. The statistics based on raw data are reported in **Supplementary Table 1**. SBP, systolic blood pressure; DBP, diastolic blood pressure; BMI, body mass index; WC, waist circumference; FM, fat mass; FFM, free fat mass; FPG, fasting blood glucose; HbA1c, glycated hemoglobin; HOMA, Homeostatic Model Assessment for Insulin Resistance; HDL, High-Density Lipoprotein; LDL, low-density lipoprotein; LH, Luteinizing hormone; FSH, Follicle-stimulating hormone; FAI, Free androgen index; DHEAS, Dehydroepiandrosterone sulfate; AMH, Anti-müllerian hormone; V, volume.

A ROC curve analysis (**Figure 1**) was performed and showed that SHBG has an accuracy of 81.1% (69.1-93.0, p <0.0001) in identifying women with a pathological NAFLD-LFS. In particular, a value of 33.4 nmol/l was found to be the best cut-off, with a sensitivity of 73.3% and a specificity of 70.7%. Using this threshold, the probability of having a pathological NAFLD-LFS score in women with SHBG <33.4 or >= 33.4 nmol/L was 47.8% and 12.1%, respectively (p=0.003). Subsequently, patients were categorized according to this SHBG threshold and the differences between patients with SHBG above or below 33.4 nmol/L in metabolic, hormonal and ultrasound parameters were evaluated (**Figure 2** and **Supplementary Table 1** **)**. At univariate analysis, values of SHBG<33.4 nmol/l were positively associated with several cardio-metabolic risk factors [SBP, DBP, BMI, waist circumference, (FM), Free Fat Mass (FFM), insulin, HOMA index and triglycerides], and with androstenedione. **Figure 2** also reports the clear-cut and obvious association between SHBG and FAI, a parameter resulting from the TT to SHBG ratio. On the contrary, a negative association was found between SHBG and HDL cholesterol levels.

In order to further validate the identified SHBG cut-off, we retested the above-mentioned relationships in an independent cohort of women seeking medical care for FSD at the same outpatient clinic (cohort 2).

**Table 1** also lists the main characteristics of this second cohort and the comparison with the cohort # 1.

**Figure 3:**
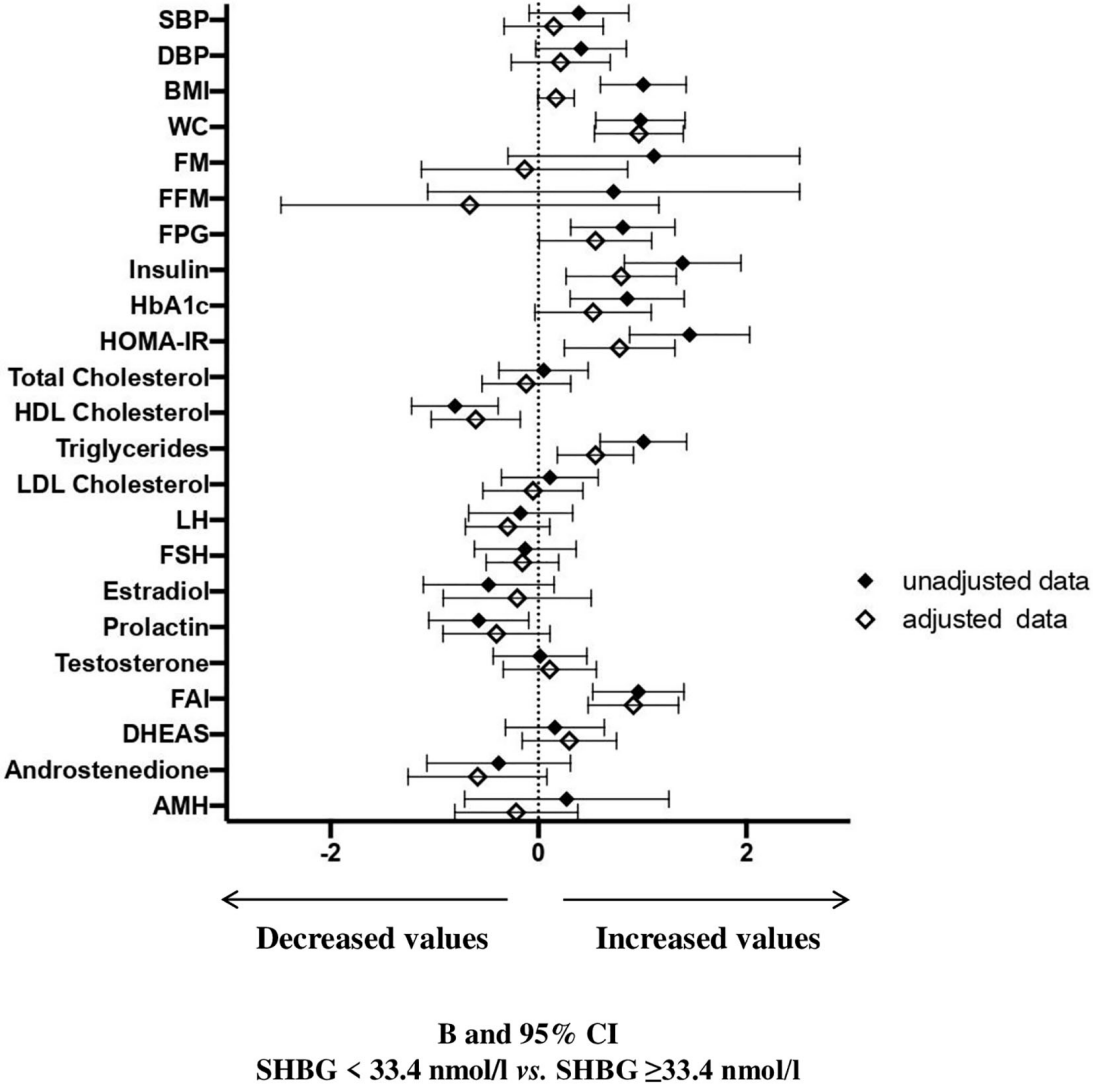
B and 95% confidence interval (CI) for several metabolic and hormonal parameters as a function of SHBG <33.4 nmol/L as compared with SHBG ≥33.4 nmol/L in Cohort 2. Data are expressed as number of standard deviations from the mean value. The standardized values are based on log-transformed parameters. The statistics based on raw data are reported in **Supplementary Table 2**. Black diamonds: unadjusted data; white diamonds: data adjusted for age and waist circumference. SBP, systolic blood pressure; DBP, diastolic blood pressure; BMI, body mass index; WC, waist circumference; FM, fat mass; FFM, free fat mass; FPG, fasting blood glucose; HbA1c, glycated hemoglobin; HOMA, Homeostatic Model Assessment for Insulin Resistance; HDL, High-Density Lipoprotein; LDL, low-density lipoprotein; LH, Luteinizing hormone; FSH, Follicle-stimulating hormone; FAI, Free androgen index; DHEAS, Dehydroepiandrosterone sulfate; AMH, Anti-müllerian hormone; V, volume.

At univariate analysis (**Figure 3** and **Supplementary Table 2**), women with SHBG <33.4 nmol/l had significantly higher BMI, waist circumference, fasting blood glucose, insulin, glycated hemoglobin, HOMA index, triglycerides, prolactin, FAI and lower HDL cholesterol. After adjusting for age and waist circumference, the associations among insulin, HOMA index, triglycerides, HDL cholesterol and FAI still retained statistical significance. In addition, also the association between SHBG levels and waist circumference retained significance, in an age-adjusted model. Women with SHBG <33.4 nmol/l had also a significantly higher NAFLD-LFS, both in unadjusted and adjusted models (p<0.0001 and p=0.001, respectively – data shown in **Supplementary Tables**).

**Figure 4:**
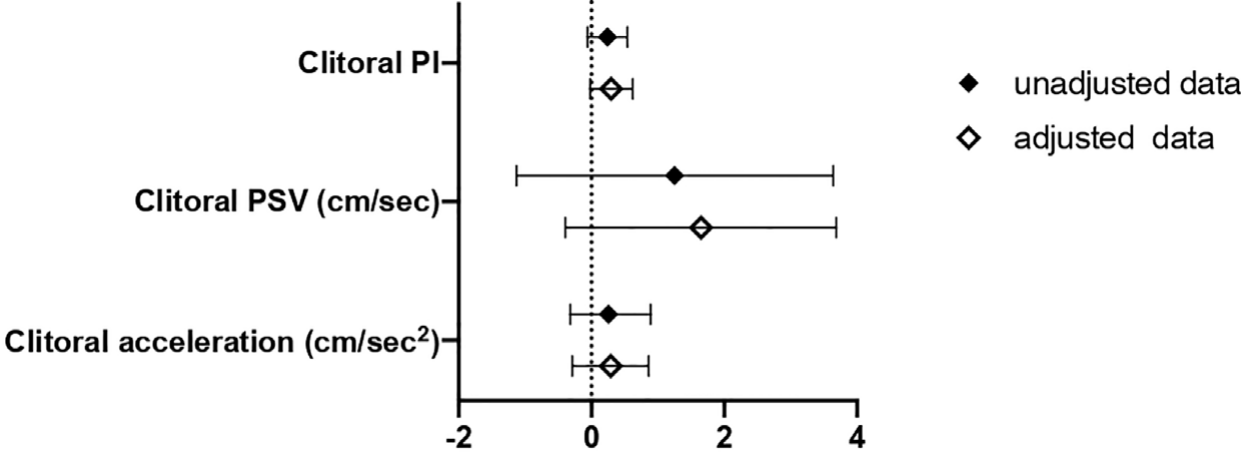
B and 95% confidence interval (CI) for clitoral vascular parameters as a function of SHBG<33.4 nmol/L as compared with SHBG≥33.4 nmol/L in Cohort 2. Data are expressed as number of standard deviations from the mean value. Black diamonds: unadjusted data; white diamonds: data adjusted for age, menopausal status and years from menopause. PI, pulsatility index; PSV, peak systolic velocity; ACC, basal acceleration.

In this second cohort, the association between SHBG values and clitoral ultrasound parameters was also evaluated (**Figure 4**). We observed a higher clitoral pulsatility index in women with SHBG < 33.4 nmol/l, even though a full statistical significance was not achieved.

In order to assess which SHBG value may be considered as “normal”, women from cohort #2 of childbearing age and not affected by MetS (n = 86) were selected. In this population, the median SHBG value was 71.55 nmol/l with 90% of the population ranging from 39.2 to 166.7 nmol/L. Therefore, the value of 33.4 nmol/l, identified in women with hyperandrogenism and/or oligomenorrhea, may be considered below normality for healthy women of childbearing age.

## Discussion

The present study confirms the correlation between NAFLD and metabolic parameters in a cohort of patients with oligomenorrhea and/or hirsutism and identify a cut-off of SHBG able to predict the risk of NAFLD in this population. Young women with SHBG below 33.4 nmol/L showed a higher probability of having pathological NAFLD-LFS score as compared to those with SHBG above this value. In an independent population of patients seeking medical care for sexual dysfunction, this cut-off was also able to discriminate women at higher cardio-metabolic risk. By analyzing in the first cohort the correlations between the NAFLD-LFS and several parameters, positive correlations were found with blood pressure, glyco-lipidic parameters, inflammatory indices, waist circumference and BMI, while a negative correlation was found with SHBG. The latter result confirms previously reported data, which identify SHBG as an indicator of liver metabolic impairment (55).

SHBG is a glycoprotein produced by the liver and it is involved in the transport of sex hormones in the bloodstream, having a major role in the regulation of their circulating free levels. Interestingly, this protein is increasingly recognized as a hepatokine and is involved in the occurrence and development of metabolic disorders and of their cardiovascular consequences (56, 57).

So far, the relationship between SHBG and NAFLD in patients with PCOS has been evaluated in a single retrospective study. As compared with age- and BMI-matched control women, PCOS patients had higher serum testosterone and reduced SHBG levels, being both associated with an increased NAFLD hazard (58). Interestingly, this study also showed that SHBG <30 nmol/L was associated with five-fold higher NAFLD risk than SHBG >60 nmol/L (58). This previous finding corroborates our results that, obtained with a different approach, identified a SHBG threshold of 33.4 nmol/L as the most appropriate to discriminate women with pathologic NAFLD-LFS.

In the first study cohort, including women with oligomenorrhea and/or hirsutism and enriched in PCOS patients, the assessment of metabolic parameters confirmed that SHBG <33.4 nmol/L identifies patients with adverse metabolic profile, including worse blood pressure, BMI, waist circumference, body composition (increased fat mass and reduced fat free mass), insulin, triglycerides and HDL cholesterol.

In order to confirm the reliability of SHBG <33.4 nmol/L as a marker of metabolic impairment and increased NAFLD risk, a second larger independent cohort of women with sexual dysfunction was analyzed. Also in this population, SHBG values lower than 33.4 nmol/l allowed to identify women with higher BMI and waist circumference, worse glyco-lipid profile and, notably, pathological NAFLD-LFS values. Furthermore, in this population, SHBG values lower than 33.4 nmol/l, independently of age, menopausal status and years since menopause, also identified patients with a numerical higher clitoral PI. This is in line with recent findings, which showed that, in women consulting for sexual dysfunction, the clitoral PI, an index of vascular resistance, is higher in patients with metabolic impairments (59–61).

Hence, SHBG <33.4 nmol/L is an effective marker for identifying women with altered metabolic parameters and higher NAFLD risk in either high risk groups, such as those with PCOS, or those consulting for conditions not directly linked to metabolic disease, such as female sexual dysfunction.

The role of SHBG as a marker of metabolic alterations is not completely understood. Some preclinical studies have been aimed at investigating whether reduced SHBG is a cause or a consequence of the metabolic dysregulation. Overexpression of SHBG, by creating a double transgenic mouse (SHBG-C57BL/ksJ-db/db), in a NAFLD model or in a diet–induced model of hepatic steatosis, significantly reduced liver fat accumulation through PPARγ modulation (62). On the contrary, an increased hepatic lipogenesis and pro-inflammatory cytokines secretion downregulates SHBG production (63). These data provide potential mechanisms by which SHBG may be either a cause or a consequence of NAFLD onset and progression (57). Clinical data also support a putative causal role of SHBG towards the development of NAFLD. In fact, in a recent study in a cohort of 3389 Chinese patients, lower baseline SHBG was associated with a higher occurrence of NAFLD, during a 3-year follow-up, and, conversely, higher SHBG at study entry predicted a more frequent recovery from NAFLD (64). However, due to the sex steroid-dependence of SHBG, its own role in describing the metabolic status is largely unclear. In men, it has been recently shown that SHBG, independently of T, is associated with worse lipid profile and blood pressure (65), thus supporting the value of SHBG as a pure marker of metabolic disorders. In women, increased testosterone levels are associated with lower SHBG, as well as with dyslipidemia and insulin resistance (66, 67). Indeed, this complicates the interpretation of lower SHBG levels. Interestingly, in both the experimental cohorts evaluated in the present study, SHBG below 33.4 nmol/L did not identify women with increased androgen levels, thus excluding that these hormones participate in worse metabolic pattern associated with low SHBG, at least when this threshold is used.

The relevance of our results relies on the recognition of SHBG as a single, and easy-to-obtain, parameter that can reliably identify patients with more adverse metabolic parameters and, even more importantly, establish which patients have higher probability of having NALFD. This may simplify the every-day clinical practice because, despite being a streamlining, the available scores take extra time during the visit for their calculation. For this reason, in a clinical setting, having a single, rapid, and low cost marker, such as SHBG, can be the first “alarm bell” to deepen liver conditions in these patients (55, 68). The confirmation of the reliability of this value as a cut-off indicative of a higher risk of NAFLD even in an independent and population with different clinical background, strengthen the generalizability of our results.

The systematic assessment of NAFLD risk is of pivotal importance because NAFLD is associated with an increased prevalence and incidence of T2DM and cardiovascular disease (69–71). The recognition of patients that, although young or apparently healthy, have hallmarks of NAFLD disease may help the physician in directing the diagnostic workup and further investigate liver disease thorough more accurate, albeit more expensive and invasive, tests and to implement more strict lifestyle or pharmacologic interventions.

This study has some limitations, including the small sample size and the heterogeneity of the cohort #1, which is not purely made of PCOS patients but it includes women with clinical features that deserve periodic assessment for a possible development of PCOS. In fact, it is known that subjects with even a single diagnostic factor are at higher risk of developing PCOS as well as MetS and NAFLD (72, 73). In addition, NAFLD was not diagnosed with imaging studies but only estimated with a clinical algorithm. Finally, in both the cohorts, androgens were not measured by the gold-standard method, i.e. mass spectrometry, although a highly reliable immunoassay has been used.

The strength of this study is that the threshold value of SHBG that we analytically found in this small sample replicates values previously identified in a larger population. In our analytical sample, SHBG <33.4 nmol/L has good accuracy, sensitivity and specificity, and it is capable of identifying higher risk of NAFLD, either in a population of young women predisposed to metabolic impairment or in young-adult/middle-aged women consulting for female sexual dysfunction.

A future perspective will be to evaluate whether treating NAFLD with lifestyle interventions (nutrition and physical activity) or with medications has consequences on SHBG values, thus confirming its role as a marker of liver health.

In conclusion, this study provides a new evidence in the complex diagnostic process of NAFLD in patients at a higher risk, which can be used as a first test to calibrate a subsequent targeted diagnostic assessment.

## Data Availability Statement

The original contributions presented in the study are included in the article/**Supplementary Material**. Further inquiries can be directed to the corresponding author.

## Ethics Statement

The studies involving human participants were reviewed and approved by the Ethics Committee “Area Vasta Centro.” Written informed consent to participate in this study was provided by the participants or by participants’ legal guardian/next of kin.

## Author Contributions

VDS, GR, and LV conceptualized and designed the study. VDS acquired the data. VDS, EM, GR, and LV analyzed and interpreted the data. VS and EM drafted the article. VDS and LV revised the article for intellectual content. All authors contributed to the article and approved the submitted version.

## Funding

This work was supported by the unrestricted Grant by Theramex Italy.

## Conflict of Interest

The authors declare that this study received funding from Theramex Italy. The funder was not involved in the study design, collection, analysis, interpretation of data, the writing of this article or the decision to submit it for publication.
