# Supplementary material for: SHBG as a Marker of NAFLD and Metabolic Impairments in Women Referred for Oligomenorrhea and/or Hirsutism and in Women With Sexual Dysfunction
Source: Front Endocrinol (Lausanne). 2021 Mar 29;12:641446. doi: 10.3389/fendo.2021.641446 (PMC8040974; doi:10.3389/fendo.2021.641446)
Supplement: Supplementary file 1 [file Table_1.docx]

| **SHBG <33.4 nmol/l vs SHBG ≥33.4 nmol/l** | | |
| --- | --- | --- |
|  | **Univariate analysis *** | |
|  | **B**  **[95% IC]** | **P value** |
| **SBP (mm Hg)** | 6.92  [0.64 ─13.19] | **0.031** |
| **DBP (mm Hg)** | 4.82  [0.33 ─ 9.30] | **0.036** |
| **BMI (kg/m^2^)** | 7.00  [4.27 ─ 9.74] | **<0.0001** |
| **Waist circumference (cm)** | 17.68  [9.95 ─ 25.41] | **<0.0001** |
| **FM (kg)** | -8.16  [-14.8 ─ -1.51] | **0.018** |
| **FFM (kg)** | -2.02  [-5.48 ─ 1.43] | 0.243 |
| **FPG (mg/dl)** | 3.76  [-1.03 ─ 8.54] | 0.122 |
| **Insulin (mU/L)** | 9.46  [5.63 ─ 13.30] | **<0.0001** |
| **Glycated hemoglobin (mmol/mol)** | -0.56  [-1.97 ─ 0.86] | 0.434 |
| **HOMA index** | 2.28  [1.27 ─ 3.30] | **<0.0001** |
| **Total cholesterol (mg/dl)** | 2.72  [-12.37 ─ 17.81] | 0.720 |
| **HDL cholesterol (mg/dl)** | -8.84  [-15.78 ─ -1.90] | **0.013** |
| **Triglycerides**  **(mg/dl)** | 28.83  [11.04 ─ 46.61] | **0.002** |
| **LDL cholesterol (mg/dl)** | 1.14  [-12.55 ─ 14.84] | 0.868 |
| **LH (U/L)** | 0.53  [-2.76 ─ 3.83] | 0.748 |
| **FSH (U/L)** | 0.25  [-0.72 ─ 1.22] | 0.606 |
| **Estradiol (pg/ml)** | -4.25  [-18.35 ─ 9.85] | 0.548 |
| **Prolactin (ng/ml)** | 0.17  [-2.75 ─ 3.08] | 0.910 |
| **Testosterone (nmol/L)** | 0.09  [-0,37 ─ 0,54] | 0.707 |
| **FAI** | 5.51  [3.26 ─ 7.76] | **<0.0001** |
| **Androstenedione (nmol/L)** | 2.38  [0.33 ─ 4.43] | **0.023** |
| **DHEAS (µmol/L)** | 0.44  [-0,82 ─ 1,71] | 0.489 |
| **AMH (ng/ml)** | 1.56  [-1.03 ─ 4.15] | 0.231 |
| **Right ovarian volume (cc)** | 0.67  [-2.04 ─ 3.39] | 0.620 |
| **Left ovarian volume (cc)** | -0.15  [-4.27 ─ 3.97] | 0.943 |

**Supplementary table 1.**

**Associations between SHBG values (cut-off 33.4 nmol/L) and clinical, metabolic, hormonal and ultra-sonographic parameters**

***** **Unadjusted correlation coefficients (B) and levels of significance (P) were derived from linear regression analysis**

**SBP= Systolic Blood Pressure; DBP= Diastolic Blood Pressure; BMI=Body Mass Index; FM= Fat Mass; FFM= Free Fat Mass; FPG= Fasting Plasma Glucose; LDL= Low Density Lipoprotein; HDL= High Density Lipoprotein; LH= Luteinizing Hormone; FSH= Follicle Stimulating Hormone; SHBG=Sex Hormone Binding Globulin; FAI= Free Androgen Index; DHEAS= Dehydro-epiandrosterone Sulfate; AMH= Anti Mullerian Hormone**

| **SHBG <33.4 nmol/l vs SHBG ≥33.4 nmol/l** | | | | |
| --- | --- | --- | --- | --- |
|  | **Univariate analysis*** | | **Multivariate analysis°** | |
|  | **B**  **[95% IC]** | **P value** | **B**  **[95% IC]** | **P value** |
| **SBP (mm Hg)** | 5.28  [-1.57 ─ 12.13] | 0.129 | 1.91  [-4.87 ─ 8.70] | 0.578 |
| **DBP (mm Hg)** | 3.65  [-0.15 ─ 7.44] | 0.060 | 1.87  [-2.28 ─ 6.03] | 0.374 |
| **BMI (kg/m^2^)** | 6.40  [3.62 ─ 9.19] | **<0.0001** | 0.73  [-0.44 ─ 1.90] | 0.218 |
| **Waist circumference (cm)** | 15.54  [8.53 ─ 22.54] | **<0.0001** | 15.37  [8.37 ─ 22.37] | <**0.0001 ^#^** |
| **FM (%)** | 9.18  [-1.82 ─ 20.17] | 0.098 | -0.49  [-7.25 ─ 6.27] | 0.881 |
| **FFM (%)** | -6.78  [-22.43 ─ 8.88] | 0.379 | 2.60  [-6.60 ─ 11.81] | 0.561 |
| **FPG (mg/dl)** | 0.18  [0.05 ─ 0.30] | **0.008** | 0.12  [-0.02 ─ 0.26] | 0.090 |
| **Insulin (mU/L)** | 15.06  [9.11 ─21.01] | **<0.0001** | 9.48  [3.91 ─ 15.06] | **0.001** |
| **Glycated hemoglobin (mmol/mol)** | 6.88  [2.37 ─ 11.38] | **0.003** | 4.30  [-0.36 ─ 8.97] | 0.070 |
| **HOMA index** | 4.70  [2.70 ─ 6.69] | **<0.0001** | 2.90  [0.95 ─ 4.85] | **0.004** |
| **Total cholesterol (mg/dl)** | 2.42  [-15.95 ─20.78] | 0.795 | -4.07  [-22.66 ─ 14.51] | 0.665 |
| **Triglycerides (mg/dl)** | 56.89  [30.81 ─ 82.97] | **<0.0001** | 31.57  [6.92 ─ 56.21] | **0.013** |
| **LDL cholesterol (mg/dl)** | 2.45  [-13.93 ─ 18.82] | 0.768 | -2.96  [-20.02 ─ 14.11] | 0.732 |
| **HDL cholesterol (mg/dl)** | -12.44  [-19.16 ─ -5.72] | **<0.0001** | -9.08  [-16.04 ─ -2.11] | **0.011** |
| **NAFLD-LFS** | 5.88  [3.63 ─ 8.13] | **<0.0001** | 4.18  [2.05 ─ 6.31] | **0.001** |
| **LH (U/L)** | -3.99  [-12.97 ─ 4.99] | 0.381 | -6.22  [-14.79 ─ 2.35] | 0.153 |
| **FSH (U/L)** | -8.15  [-28.06 ─ 11.75] | 0.419 | -5.85  [-22.84 ─ 11.15] | 0.497 |
| **Estradiol (pmol/l)** | -11.57  [-71.23 ─ 48.09] | 0.700 | 43.29  [-16.69 ─ 103.27] | 0.154 |
| **Prolactin (mU/L)** | -92.31  [-180.73 ─ -3.88] | **0.041** | -63.28  [-160.54 ─ 33.98] | 0.200 |
| **Testosterone (nmol/L)** | 0.71  [-0.86 ─ 2.27] | 0.373 | 1.21  [-0.34 ─ 2.76] | 0.126 |
| **FAI** | 6.86  [2.48 ─ 11.24] | **0.002** | 7.35  [2.93 ─ 11.77] | **0.001** |
| **Androstenedione (nmol/L)** | -1.10  [-2.93 ─ 0.73] | 0.233 | -1.77  [-3.59 ─ 0.05] | 0.056 |
| **DHEAS (µmol/L)** | 0.03  [-0.97 ─ 1.03] | 0.955 | 0.43  [-0.51 ─ 1.37] | 0.369 |
| **AMH (ng/ml)** | 0.41  [-1.77 ─ 2.60] | 0.706 | -0.40  [-2.12 ─ 1.32] | 0.642 |

**Supplementary Table 2**

**Associations between SHBG levels (cut-off 33.4 nmol/l) and clinical, metabolic, inflammatory and hormonal parameters in women with FSD**

*** Unadjusted correlation coefficients (B) and levels of significance (P) were derived from linear regression analysis**

**° Adjusted correlation coefficients (B) and levels of significance (P) were derived from linear regression analysis after adjustment for age and waist circumference**

**_#_ Adjusted for age**

**SBP= Systolic Blood Pressure; DBP= Diastolic Blood Pressure; BMI=Body Mass Index; FM= Fat Mass; FFM= Free Fat Mass; FPG= Fasting Plasma Glucose; LDL= Low Density Lipoprotein; HDL= High Density Lipoprotein; LH= Luteinizing Hormone; FSH= Follicle Stimulating Hormone; FAI= Free Androgen Index; DHEAS= Dehydro-epiandrosterone Sulfate; AMH= Anti Mullerian Hormone**
